# MACC1 promotes pancreatic cancer metastasis by interacting with the EMT regulator SNAI1

**DOI:** 10.1038/s41419-022-05285-8

**Published:** 2022-11-04

**Authors:** Xianglian Zhang, Ya Luo, Yu Cen, Xin Qiu, Jing Li, Mengmeng Jie, Shiming Yang, Shanyu Qin

**Affiliations:** 1 Department of Gastroenterology, The First Affiliated Hospital of Guangxi Medical University, Nanning, 530021 China; 2 Department of Gastroenterology, Xinqiao Hospital, Third Military Medical University, Chongqing, 400037 China

**Keywords:** Oncogenes, Metastasis

## Abstract

Metastasis is the dominant cause of cancer-related mortality. Metastasis-associated with colon cancer protein 1 (MACC1) has been proven to play a critical role in cancer metastasis. However, the prometastatic role of MACC1 in regulating the pancreatic cancer (PC) metastatic phenotype remains elusive. Here, we report that MACC1 is highly expressed in The Cancer Genome Atlas (TCGA) and tissue microarray (TMA) and identified as a good indicator for poor prognosis. Overexpression or knockdown of MACC1 in PC cells correspondingly promoted or inhibited pancreatic cancer cell migration and invasion in a MET proto-oncogene receptor tyrosine kinase (MET)-independent manner. Notably, knockdown of MACC1 in PC cells markedly decreased the liver metastatic lesions in a liver metastasis model. Mechanistically, MACC1 binds to the epithelial-mesenchymal transition (EMT) regulator snail family transcriptional repressor 1 (SNAI1) to drive EMT via upregulating the transcriptional activity of SNAI1, leading to the transactivation of fibronectin 1 (FN1) and the trans-repression of cadherin 1 (CDH1). Collectively, our results unveil a new mechanism by which MACC1 drives pancreatic cancer cell metastasis and suggest that the MACC1-SNAI1 complex-mediated mesenchymal transition may be a therapeutic target in pancreatic cancer.

## Introduction

Pancreatic cancer (PC), with one of the lowest 5-year survival rates, is known to be the most frequent type of solid cancer worldwide, ranking fourth in causing cancer death [1]. In addition, the incidence of PC continues to increase in both men and women. To some extent, PC constitutes a major public health problem affecting human health worldwide [2]. High rates of metastasis are the main reason for the poor clinical outcome of patients with PC [3]. At the time of cancer diagnosis, ~50% of PC cases are in the metastatic form with an average survival of less than a year [4]. Thus, elucidating the molecular mechanisms mediating PC cell invasion and metastasis is essential to improve therapeutic outcomes. As first reported in 2008 by Stein et al. [5], MACC1 acts as a vital transcriptional regulator of the metastasis-inducing hepatocyte growth factor (HGF)/Met pathway in colon cancer, predicting the risk of metastasis in early cancer stages. Subsequently, MACC1 has been further regarded as a prognostic biomarker for multiple solid cancers, such as pancreatic, esophageal, gastric, lung, hepatobiliary, breast, ovarian, nasopharyngeal, and renal cancers and glioblastomas, and its expression level is positively associated with tumor progression, metastasis development and patient survival [6–12]. A comparative study demonstrated that inhibiting MACC1 expression restricts tumor progression and metastasis [13]. However, the role of MACC1 in PC has seldom been reported, and its exact underlying molecular mechanism remains elusive. This prompted us to investigate the precise role of MACC1 in PC. In our study, MACC1 was aberrantly overexpressed in human PC tissues compared with adjacent normal pancreatic tissues and was strongly correlated with metastasis and poor prognosis. In addition, MACC1 could be involved in the promotion of a metastatic phenotype both in vitro and in vivo, unexpectedly independent of activating the HGF/c-Met pathway. Here, we also aimed to identify novel transcriptional target genes of MACC1 by screening for metastasis-related gene expression. For further analysis, we focused on the Fibronectin 1 (*FN1*) gene, which was obviously upregulated in PC tissues and significantly correlated with metastasis in human PC specimens. *FN1*, encoding an extracellular matrix protein, exerts its oncogenic role by modulating tumor extracellular matrix remodeling, motility, and metastasis in multiple solid tumors [14]. According to previous reports, multiple transcription factors (such as HSF1, EGR1, ATF3, LEF1, CREB1, SNAI1 and HMGA2) regulate FN1 expression at the transcriptional level [15–21]. After screening for these transcription factors, we demonstrated that the binding of MACC1 to SNAI1 upregulated SNAI1 transcriptional activity, which led to upregulated levels of FN1 *and* downregulated levels of CDH1, further initiating and driving the epithelial-mesenchymal transition (EMT) transcription program. In aggregate, these findings provide novel insights into the mechanisms involved in MACC1-mediated PC metastasis.

## Results

### MACC1 is highly expressed in human pancreatic cancer tissues and significantly associated with poor prognosis

**Fig. 1:**
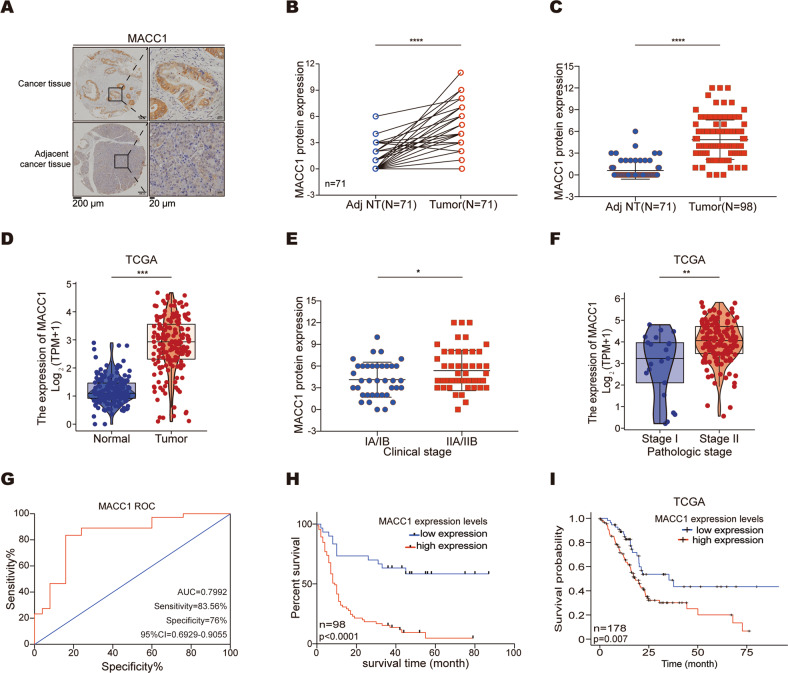
MACC1 expression is upregulated in PC tissues and correlates with poor outcomes in PC patients. **A** Representative images of immunohistochemistry for MACC1 expression levels in PC TMAs. Scale bar, left, 200 μm; right, 20 μm. **B** Comparison of MACC1 staining score in paired PC (tumor, *n* = 71) and the corresponding adjacent noncancerous tissues (Adj NT, *n* = 71). Two-tailed t test. ****p < 0.0001. **C** Comparison of MACC1 staining scores in unpaired PC (tumor, *n* = 98) and adjacent noncancerous tissues (Adj NT, *n* = 71). Two-tailed t test. ****p < 0.0001. **D** *MACC1* expression in TCGA PC and normal tissues. Two-tailed t test. ***p < 0.001. **E** MACC1 protein expression levels in PC samples grouped by clinical stage. Two-tailed t test. *p < 0.05. **F** *MACC1* mRNA expression levels in PC samples grouped by pathological stage (data retrieved from the TCGA database). Mann–Whitney test. **p < 0.01. **G** ROC analysis to evaluate the predictive value of MACC1 levels for patient survival time. **H** Kaplan–Meier curve for overall survival by MACC1 protein levels. **I** Survival plot in TCGA PC patients according to *MACC1* mRNA expression levels.

To evaluate the clinical significance of MACC1 in PC, we performed IHC staining on a tissue microarray comprising 99 PC samples (containing 71 pairs of primary tumor tissues and the corresponding adjacent noncancerous tissues). Our results showed that MACC1 protein was abundant in PC tissues (Fig. 1A) and was highly expressed in both paired (Fig. 1B) and unpaired cancer tissues (Fig. 1C) when compared to noncancerous tissues. We further compared MACC1 expression between normal and PC tissues in The Cancer Genome Atlas (TCGA). As expected, the TCGA data showed that MACC1 was highly expressed in PC tissues compared to normal tissues (Fig. 1D) and was upregulated in multiple human tumor types (Fig. S1A). Next, we investigated the clinical implications of MACC1 via analysis of the relationship between clinicopathological characteristics and MACC1 levels in the TMA and TCGA. We discovered that MACC1 was closely associated with the pathological stage (including pathological stage and advanced T classification) and clinical stage (including clinical stage and distant metastasis) of PC patients (Fig. 1E, F and Fig. S1B, C). In addition, the ROC examination indicated that MACC1 could be a good indicator of PC prognosis (Fig. 1G). Further survival analysis showed that patients in the ‘MACC1-high’ group had significantly shorter overall survival than those in the ‘MACC1-low’ group (Fig. 1H). The results were consistent with the data from the TCGA database (Fig. 1I). Thus, MACC1 is clinically relevant in PC.

### MACC1 facilitates PC cell migration and invasion in a MET-independent manner

**Fig. 2:**
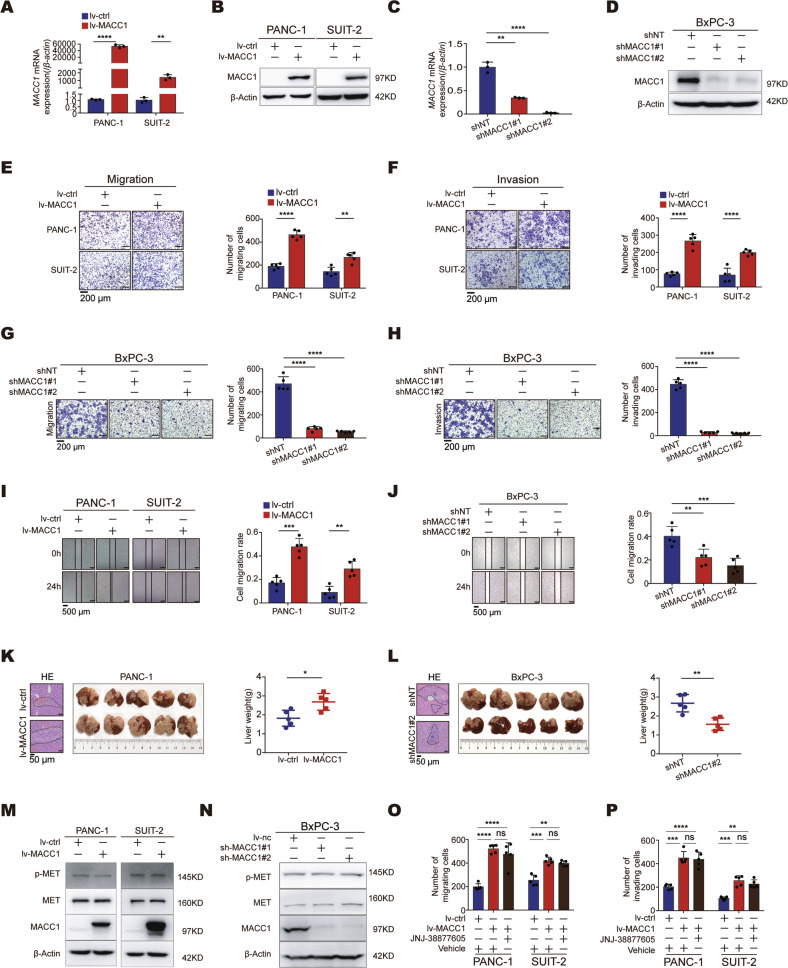
MACC1 drives PC cell migration and invasion independent of MET. **A** Overexpression of *MACC1* (lv-MACC1) in PANC-1 and SUIT-2 cells as determined by qRT–PCR. **p < 0.01, ****p < 0.0001. **B** Overexpression of MACC1 in PANC-1 and SUIT-2 cells as determined by Western blot. **C** Knockdown of *MACC1* by two independent shRNAs (shMACC1#1, shMACC1#2) in BxPC-3 cells as determined by qRT–PCR. **p < 0.01, ****p < 0.0001. **D** Knockdown of MACC1 by two independent shRNAs in BxPC-3 cells as determined by Western blot. **E, F** The effect of MACC1 overexpression on PANC-1 and SUIT-2 cells migration and invasion. Scale bars, 200 μm. **p < 0.01, ****p < 0.0001. **G, H** The effect of MACC1 knockdown on BxPC-3 cells migration and invasion. Scale bars, 200 μm. ****p < 0.0001. **I** Scratch assay following MACC1 overexpression in PANC-1 and SUIT-2 cells. Scale bars, 500 μm. **p < 0.01, ***p < 0.001. **J** Scratch assay following MACC1 knockdown in BxPC-3 cells. Scale bars, 500 μm. **p < 0.01, ***p < 0.001. **K** PANC-1 cells that were infected with either MACC1-expressing (lv-MACC1) or control virus (lv-ctrl) were implanted into randomized athymic nude mice by spleen injection (five mice per group). Representative images of HE-stained sections in dissected livers one month after inoculation are shown (left). Representative images of liver metastasis are shown (middle). The metastatic burdens were quantified based on the liver weight (right). Data are shown as the mean ± SD. Scale bars, 50 μm. Two-tailed t test. *p < 0.05. **L** BxPC-3 cells stably expressing a nontarget shRNA (shNT) or shMACC1#2 were injected into randomized athymic nude mice by spleen injection (five mice per group). Representative images of HE-stained sections in dissected livers one month after inoculation are shown (left). Representative images of liver metastasis are shown (middle). The metastatic burdens were quantified based on the liver weight (right). Data are shown as the mean ± SD. Scale bars, 50 μm. Two-tailed t test. **p < 0.01. **M, N** The effect of MACC1 overexpression in PANC-1 and SUIT-2 cells or knockdown in BxPC-3 cells on p-MET and MET levels. **O, P** The migration and invasion of PANC-1 and SUIT-2 cells treated with 50 nM JNJ-38877605 and an equal volume of DMSO was used as the vehicle control. The results of quantification of the migrated and invaded cells are shown (mean ± SD, *n* = 5). Two-way ANOVA. Scale bars, 200 μm. **p < 0.01, ***p < 0.001, ****p < 0.0001. **A**, **E, F**, Data represent the mean ± SD. of three biologically independent experiments (two-tailed t test). **B**, **D**, **M, N**, Immunoblotting experiments were performed with the indicated antibodies. Data are representative of at least three independent experiments. **C**, **G, H**, **J**, Data represent the mean ± SD. of three biologically independent experiments (one-way ANOVA).

To explore the effects of the *MACC1* gene on PC progression, we started by examining the MACC1 mRNA and protein levels in normal pancreatic ductal epithelial cells and several PC cell lines. Among them, BxPC-3 cells displayed the highest expression level of MACC1, which was derived from primary pancreatic cancer [22], while PANC-1 and SUIT-2 cells exhibited the lowest expression level of MACC1 (Fig. S2A–B). We then stably overexpressed MACC1 in PANC-1 and SUIT-2 cells with low MACC1 levels via lentiviral infection, as verified by qRT–PCR and western blotting (Fig. 2A–B). We also stably transduced BxPC-3 cells with lentiviral shRNA constructs targeting MACC1 (Fig. 2C, D). Colony formation assays suggested that ectopic expression or knockdown of MACC1 did not affect PC cell clonogenicity (Fig. S2C–E). Flow cytometric analysis of apoptosis showed that MACC1 did not affect the apoptosis of PC cells. (Fig. S2F–H). Proliferation assays revealed that overexpression of MACC1 did not promote the proliferation of PC cells (Fig. S2I, J). Knockdown of MACC1 slightly impaired the proliferation of BxPC-3 cells; however, this effect not statistically significant (Fig. S2K). To confirm these findings, we further explored the effects of the *MACC1* gene on tumor growth in vivo and found that silencing MACC1 slightly impaired the growth of PC tumors (Fig. S2L, M); however, this difference not statistically significant. Therefore, we focused on whether MACC1 plays a key role in PC metastasis. Transwell assays and wound-healing assays revealed that ectopic expression of MACC1 promoted cell invasion and migration (Fig. 2E, F, I). In contrast, knockdown of MACC1 remarkably attenuated cell migration and invasion (Fig. 2G, H, J). In addition, to further clarify the role of MACC1 in PC metastasis in vivo, we generated a liver metastasis model. After overexpressing MACC1 in PANC-1 cells via lentivirus infection, cells had a stronger ability to metastasize to the liver (Fig. 2K). However, injection of sh-MACC1 lentivirus-infected BxPC-3 cells into nude mice proved that MACC1 knockdown effectively decreased PC liver metastases (Fig. 2L). Collectively, these data indicated that MACC1 acts as an oncoprotein in PC cells to facilitate tumor metastasis.

Since a previous study demonstrated that *MET* is a key transcriptional target gene of MACC1 in multiple cancers and contributes to tumor progression [5, 11, 12, 23–28], we wanted to determine whether MACC1 plays a critical role in PC metastasis via MET. Intriguingly, we found that overexpressing MACC1 in PC cells did not affect MET mRNA or protein levels, and vice versa (Fig. S3A, B and Fig. 2M, N). This finding indicates that MACC1 mediated metastasis in PC might have different mechanisms. To further confirm these results, the human colon cancer SW480 cells and SW620 cells served as positive controls, as MACC1 was reported to be a master regulator of MET in these cells [5]. Consistent with previous findings, an increase in MACC1 levels significantly increased MET protein in SW480 cells, and a reduction in MACC1 levels resulted in a concomitant decrease in MET protein in SW620 cells (Fig. S3C, D). Finally, to further verify whether the tumor invasiveness promoted by MACC1 was dependent on MET, we treated PC cells with JNJ-38877605, a selective inhibitor of MET. Indeed, JNJ-38877605 significantly inhibited phosphorylated MET (p-MET) levels (Fig. S3E, F). Nevertheless, the inhibitor failed to reverse MACC1-mediated cell migration and invasion (Fig. 2O, P and Fig. S3G, H). These results indicated that MACC1 enhances PC cell migration and invasion in a MET-independent manner.

### MACC1 promotes PC cell metastasis by upregulating FN1

**Fig. 3:**
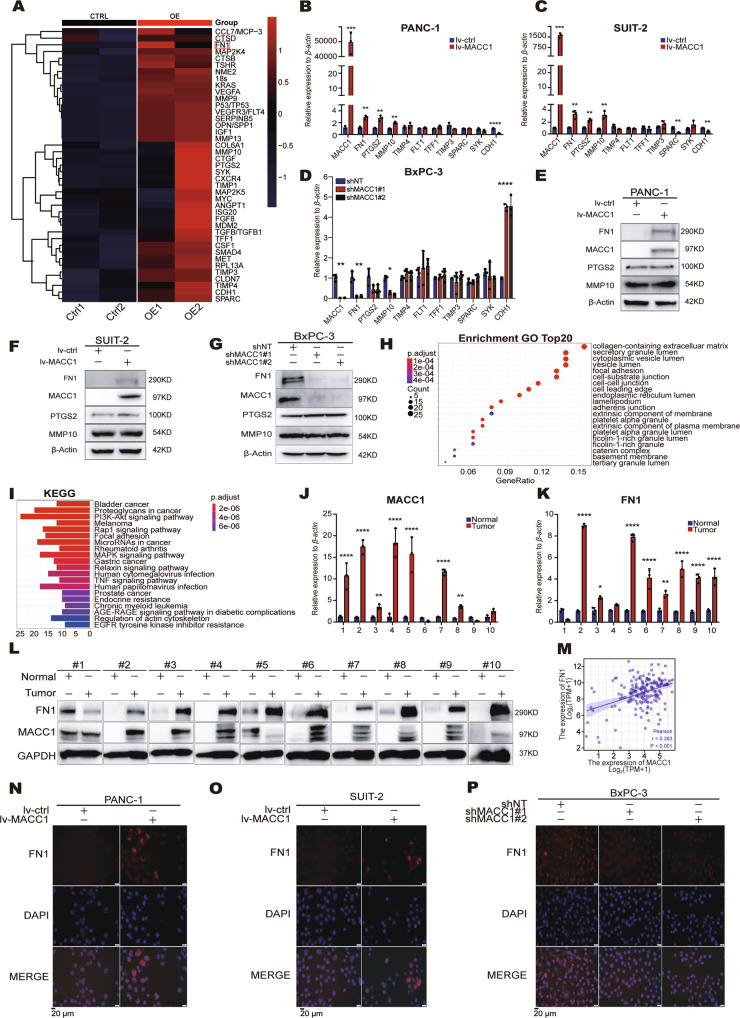
MACC1 promotes PC cell migration and invasion by upregulating FN1. **A** Heatmap of the upregulated gene expression clusters in control (Ctrl) and MACC1-overexpressing (OE) SUIT-2 cells. **B, C** Quantitative real-time PCR validation of selected differentially regulated genes after overexpression of *MACC1* in PANC-1 and SUIT-2 cells. **p < 0.01, ***p < 0.001, ****p < 0.0001. **D** Quantitative real-time PCR validation of selected differentially regulated genes after knockdown of *MACC1* in BxPC-3 cells. *p < 0.05, **p < 0.01, ****p < 0.0001. **E**–**G** Western blot for FN1, PTGS2 and MMP10 after overexpression of MACC1 in PANC-1 and SUIT-2 cells or knockdown of MACC1 in BxPC-3 cells. **H, I** GO analyses (**H**) and KEGG pathway analyses (**I**) of the upregulated genes in MACC1 overexpressing cells. **J, K** *MACC1* and *FN1* mRNA levels in primary tumor tissues and matched adjacent normal tissues from 10 PC patients. *p < 0.05, **p < 0.01, ****p < 0.0001. **L** MACC1 and FN1 protein levels in primary tumor tissues and matched adjacent normal tissues from 10 PC patients. **M** Correlation between *MACC1* and *FN1* mRNA levels in PC tissues from the TCGA datasets. R, Pearson correlation coefficient. **N**–**P** Immunofluorescence staining was performed in PANC-1, SUIT-2 and BxPC-3 cells using an anti-FN1 antibody. Representative images of immunofluorescent staining in PANC-1 and SUIT-2 cells stably expressing empty vector (lv-ctrl) or MACC1 (lv-MACC1) or in BxPC-3 cells stably expressing shNT, shMACC1#1 or shMACC1#2. Data are representative of at least three independent experiments. Scale bars, 20 μm. **D**–**F**, **J, K**, Data represent the mean ± SD. of three biologically independent experiments (two-tailed t test). **G**–**I**, **L**, Immunoblotting experiments were performed with the indicated antibodies. Data are representative of at least three independent experiments.

To further explore the mechanism underlying MACC1 promoting PC metastasis, we overexpressed MACC1 in SUIT-2 cells and performed a metastasis qPCR array (containing 136 genes). The gene expression clusters were summarized as a heatmap (Fig. 3A and Fig. S4A), and we observed that ten genes displayed a greater than twofold upregulation (log2FC > 2). Then, we carried out qRT–PCR in three different PC cell lines to validate the expression of these genes. The results showed that the expression of these genes (*FN1*, *PTGS2* and *MMP10*) was remarkably upregulated in MACC1 overexpressing cells and correspondingly downregulated in MACC1-suppressed cells (Fig. 3B–D). However, additional studies at the protein level of these genes showed that only FN1 was concordant with the expression levels of MACC1 in three PC cell lines (Fig. 3E–G). *FN1* encodes fibronectin, which is a glycoprotein that is present not only in the plasma and extracellular matrix but also at the cell surface. Functionally, fibronectin can promote an invasive phenotype in cancer cells. In addition, Gene Ontology (GO) analysis of the upregulated genes in MACC1-overexpressing cells demonstrated that the collagen-containing extracellular matrix was the most enriched cellular component term (Fig. 3H). Kyoto Encyclopedia of Genes and Genomes (KEGG) pathway analysis indicated enrichment of pathways linked to focal adhesion (Fig. 3I). The most enriched GO terms for genes related to molecular function and biological process were receptor ligand activity and extracellular matrix organization (Fig. S4B, C). These findings provide the support that *FN1* is a target gene of MACC1.

To further substantiate this point, we subsequently examined the mRNA and protein levels of MACC1 and FN1 in ten human PC tissues and paired adjacent normal tissues. The results showed that both MACC1 and FN1 were more abundant in PC tissues than in matched normal tissue (Fig. 3J–L). In addition, Pearson correlation analyses revealed a substantial correlation between MACC1 and FN1 expression in human PC samples from the TCGA database (Fig. 3M). The immunofluorescence results further confirmed these findings (Fig. 3N–P). Overall, we identified that *FN1* as a MACC1 target gene in PC for the first time.

### MACC1 upregulates FN1 by interacting with SNAI1

**Fig. 4:**
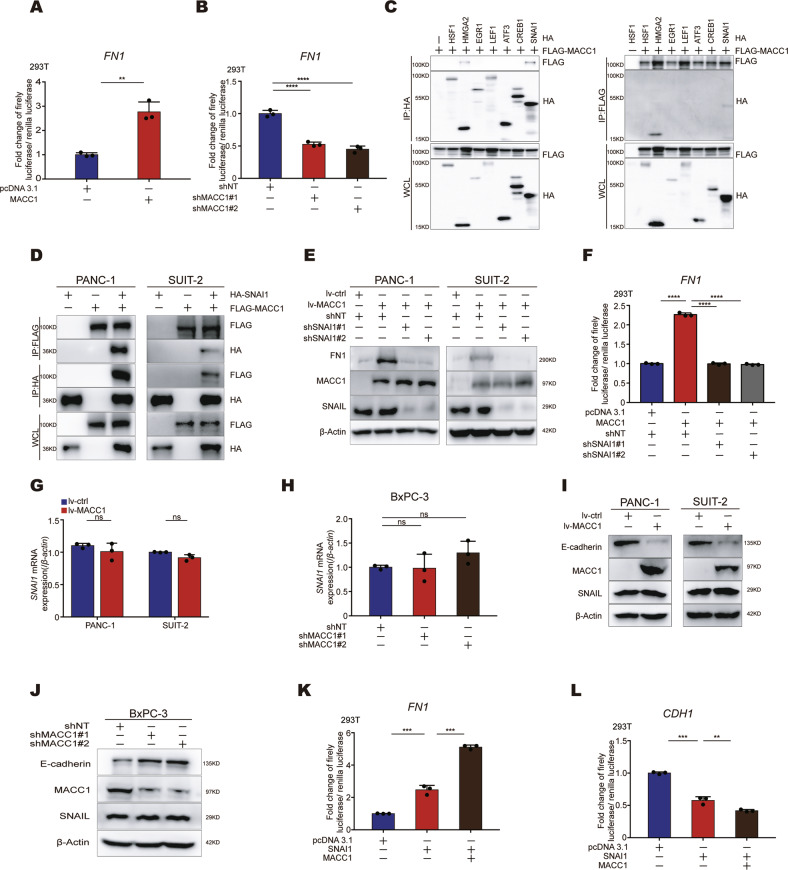
MACC1 upregulates FN1 by interacting with SNAI1. **A, B** Luciferase assay assessing *FN1* promoter activity after overexpression or knockdown of MACC1 (**B**) in 293 T cells. **p < 0.01, ****p < 0.0001. **C** Coimmunoprecipitation of exogenous HA-tagged HSF1, EGR1, ATF3, LEF1, CREB1, SNAI1 or HMGA2 with FLAG-tagged MACC1 in HEK293T cells. Eluted immunoprecipitates and whole-cell lysates (WCL) were subjected to immunoblotting analysis with anti-FLAG and anti-HA antibodies. **D** PANC-1 and SUIT-2 cells were transfected with the indicated plasmids and coimmunoprecipitation experiments between MACC1 and SNAI1 were performed. Protein samples were immunoprecipitated with anti-Flag or anti-HA beads, and then immunoprecipitates and whole-cell lysates (WCL) were immunoblotted with anti-FLAG and anti-HA antibodies. **E** Western blot for FN1, with MACC1 overexpression and SNAIL knockdown in PANC-1 and SUIT-2 cells. **F** Luciferase assay assessing FN1 promoter activity, with MACC1 overexpression and SNAIL knockdown in 293 T cells. ****p < 0.0001. **G, H** qRT–PCR analysis of SNAI1 expression with MACC1 overexpression in PANC-1 and SUIT-2 cells or knockdown in BxPC-3 (**H**) cells. ns for not significant. **I, J** Western blot for SNAIL and E-cadherin, with MACC1 overexpression in PANC-1 and SUIT-2 cells or knockdown in BxPC-3 cells. **K, L** Luciferase assay assessing *FN1* and *CDH1* promoter activity, with both MACC1 and SNAIL overexpression in 293T cells. **p < 0.01, ***p < 0.001. **A**, **G**, **K, L**, Data represent the mean ± SD. of three biologically independent experiments (two-tailed t test). **B**, **F**, **H**, Data represent the mean ± SD. of three biologically independent experiments (one-way ANOVA). **C**–**E, I**–**J**, Immunoprecipitation and immunoblotting experiments were performed with the indicated antibodies. Data are representative of at least three independent experiments.

Having revealed the regulatory relationship of MACC1 and FN1 in PC cells, we sought to interrogate the mechanism by which MACC1 regulates FN1 expression. First, we conducted a dual-luciferase reporter assay to confirm the transcriptional regulation of FN1 by MACC1. The results showed that overexpression of MACC1 increased the promoter activity of the *FN1* gene, while knockdown of MACC1 reduced the promoter activity (Fig. 4A, B). Although several downstream transcriptional target genes of MACC1 have been identified (e.g., MET, SPON2, and Nanog [5, 29, 30]), there is no clear evidence that MACC1 itself can directly bind to DNA to initiate target gene expression. In addition, the structural domain of MACC1 indicates that MACC1 may be involved in protein‒protein interactions [5, 31]. This suggests that MACC1 likely regulates the transcription of downstream genes through other proteins. For this reason, we envision that MACC1 might interact with a transcription factor to upregulate FN1 expression indirectly. It has been reported that FN1 is directly regulated by seven transcription factors (HSF1, EGR1, ATF3, LEF1, CREB1, SNAI1 and HMGA2) [15–21]. Therefore, we performed coimmunoprecipitation (Co-IP) assays to screen whether MACC1 might interact with these transcription factors. Co-IP analyses revealed that MACC1 interacts with HMGA2 and SNAI1 (which encodes SNAIL) but not HSF1, EGR1, ATF3, LEF1 or CREB1 in 293 T cells (Fig. 4C). However, only SNAI1 interacted with MACC1 (Fig. 4D), whereas HMGA2 failed to interact with MACC1 in PC cells (Fig. S5A, B). In addition, knockdown of SNAI1 in MACC1-overexpressing PC cells impaired MACC1-mediated FN1 upregulation (Fig. 4E). Furthermore, *FN1* promoter activity was strongly repressed after knockdown of SNAI1 in MACC1-overexpressing 293 T cells (Fig. 4F). These findings suggested that MACC1 interacts with SNAI1 but not with HMGA2 to upregulate FN1 expression.

Next, we sought to explore how the binding of MACC1 to SNAI1 potently triggers FN1 expression. First, we envisioned whether MACC1 affects the mRNA or protein level of SNAI1. Unexpectedly, overexpression or knockdown of MACC1 in PC cells did not affect the mRNA (Fig. 4G, H) or protein levels of SNAI1 (Fig. 4I, J). In addition, we also observed that overexpression of MACC1 promoted FN1 expression while suppressing CDH1 (which encodes E-cadherin) expression (Fig. 3B–D). SNAI1 is known to function as a transcriptional activator or transcriptional repressor for different target genes [32]. Coincidentally, *FN1* is a SNAI1-activated gene and *CDH1* is a SNAI1-repressed gene. Since MACC1 did not affect the expression of SNAI1, it is likely to regulate the transcriptional activity of SNAI1. Thus, we further examined the effect of MACC1 on SNAI1 transactivation by measuring the promoter activity of *FN1* and *CDH1* using the dual-luciferase reporter assay system. The results indicated that MACC1 augmented SNAI1-induced *FN1* promoter activity (Fig. 4K). Next, we constructed a human *CDH1* promoter (-200/+100 bp) that harbors the SNAI1 binding site E-box [33]. A dual-luciferase reporter assay revealed that MACC1 promoted SNAI1-induced *CDH1* promoter activity (Fig. 4L). In addition, overexpression of MACC1 decreased CDH1 protein levels (Fig. 4I). Conversely, MACC1 knockdown increased CDH1 protein levels (Fig. 4J). Taken together, these data indicated that MACC1 interacts with SNAI1 and promotes its transcriptional activity, thereby upregulating FN1.

### FN1 rescues PC cell migration and invasion accelerated by MACC1

**Fig. 5:**
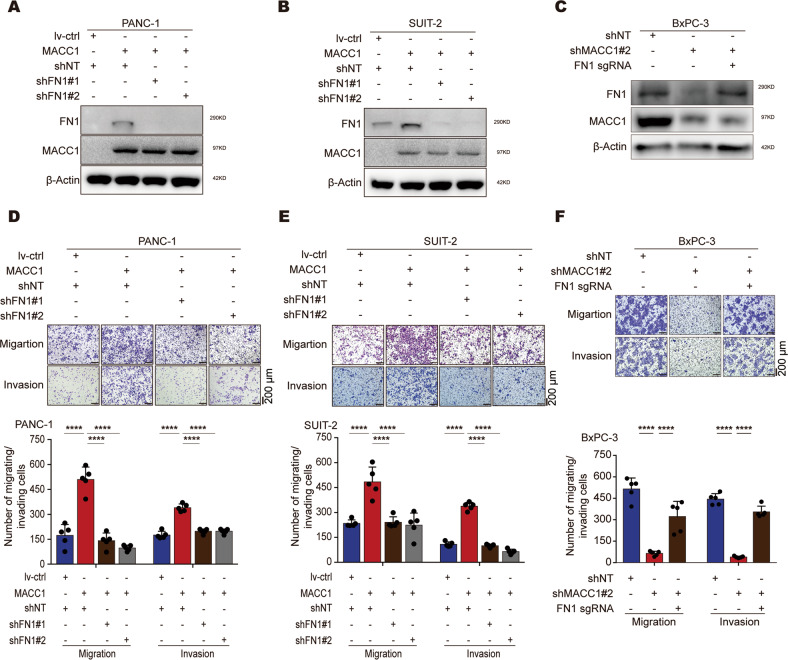
FN1 is required for MACC1-mediated enhancement of PC cell migration and invasion. **A** Western blot for FN1 in PANC-1 cells based on MACC1 overexpression and FN1 knockdown. **B** Western blot for FN1 in SUIT-2 cells based on MACC1 overexpression and FN1 knockdown. **C** Western blot for FN1 in BxPC-3 cells based on MACC1 knockdown and FN1 endogenous induction. Endogenous FN1 induction was achieved by CRISPRa. **D** Migration and invasion of PANC-1 cells based on MACC1 overexpression and FN1 knockdown. Representative images and quantification of migrated and invaded cells are shown (mean ± SD, *n* = 5). Scale bars, 200 μm. One-way ANOVA. ****p < 0.0001. **E** Migration and invasion of SUIT-2 cells based on MACC1 overexpression and FN1 knockdown. ****p < 0.0001. **F** Migration and invasion of BxPC-3 cells based on MACC1 knockdown and endogenous FN1 induction. ****p < 0.0001. **A**, **B**, **C**, Immunoblotting experiments were performed with the indicated antibodies. Data are representative of at least three independent experiments. **E**, **F**, Data represent the mean ± SD. of three biologically independent experiments. Scale bars, 200 μm. Two-tailed t test.

To test whether *FN1* is a critical downstream gene of MACC1 in regulating PC cell migration and invasion, we performed lentivirus-mediated FN1-shRNA knockdown to decrease FN1 protein levels (Fig. 5A, B). Furthermore, we induced the endogenous expression of FN1 in PC cells with stable MACC1 knockdown using a dCas9-mediated CRISPR activation system (Fig. 5C and Fig. S6A). Transwell analysis revealed that knockdown of FN1 is sufficient to abrogate MACC1-induced cell migration and invasion (Fig. 5D, E). In a reciprocal experiment, we found that overexpressing FN1 in stably infected MACC1-shRNA PC cells significantly reversed the MACC1-mediated inhibitory effect on PC cell invasion and migration (Fig. 5F). Overall, these results illustrated that FN1 is functionally essential for MACC1-mediated migration and invasion in PC cells.

### MACC1 is positively associated with FN1 expression in PC

**Fig. 6:**
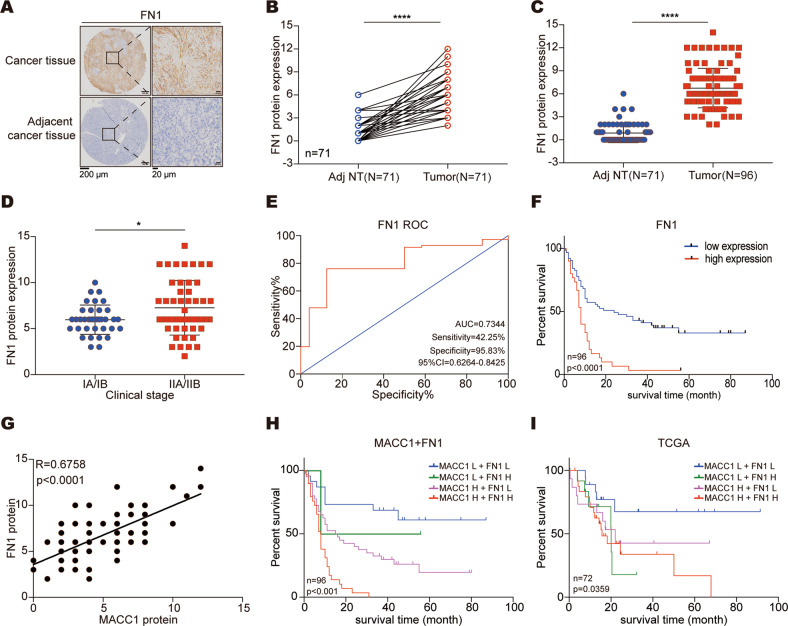
MACC1 expression level is associated with FN1 expression level in PC. **A** Representative IHC staining of FN1 in PC TMAs. Scale bar, left, 200 μm; right, 20 μm. **B** Comparison of FN1 staining scores in paired PC (tumor, *n* = 71) and the corresponding adjacent noncancerous tissues (Adj NT, *n* = 71). Two-tailed t test. ****p < 0.0001. **C** Comparison of FN1 staining scores in unpaired PC (tumor, *n* = 96) and adjacent noncancerous tissues (Adj NT, *n* = 71). Two-tailed t test. ****p < 0.0001. **D** FN1 protein expression levels in PC samples grouped by clinical stage. Two-tailed t test. *p < 0.05. **E** ROC analysis to evaluate the predictive value of FN1 for patient survival time. **F** Kaplan–Meier curve for overall survival by FN1 expression levels. **G** Correlations between MACC1 and FN1. R, Pearson correlation coefficient. **H** Combined analysis of MACC1 and FN1 expression levels in the prognostic value of patients with PC by Kaplan–Meier survival curves. **I** Survival plot in TCGA PC patients according to the combination of MACC1 and FN1 mRNA expression.

To confirm the clinical relevance of FN1, we next examined FN1 levels in a TMA and found that FN1 was noticeably elevated in PC tissues (Fig. 6A–C). In addition, FN1 was significantly related to distant metastasis (Fig. S7A) and clinical stage (Fig. 6D) in PC patients. In line with our findings, analysis of the TCGA dataset showed that FN1 expression was higher in PC tissues than in normal tissues (Fig. S7B) and was associated with the pathologic stage of PC patients (Fig. S7C). The ROC curves indicated that FN1 could be used to assess the prognosis of PC patients (Fig. 6E). Further survival analysis showed that high FN1 level was closely related to poor outcomes in PC patients (Fig. 6F). Importantly, we found that MACC1 and FN1 were closely positively correlated in the PC TMA (Fig. 6G). A positive correlation between FN1 and MACC1 was also observed in ten cancer types in TCGA (Fig. S7D). Moreover, patients with concurrent low FN1 and MACC1 levels in PC had a dramatically longer lifespan in both the TMA and TCGA (Fig. 6H–I). Collectively, these findings strongly suggest that both MACC1 and FN1 are clinically relevant in PC patients.

## Discussion

Metastasis is the leading cause of death in PC [4]. MACC1 is a metastasis-promoting factor and a prognostic biomarker for various human malignancies [34]. Here, we showed that MACC1 is overexpressed in PC, while downregulation of MACC1 in PC cells by shRNA led to inhibition of migration and invasion of PC cells. In mice, transplantation of PC cells overexpressing MACC1 caused increased metastatic burden in the liver. While MACC1 exerts its prometastatic effects by activating MET in various human cancers, including colon, gastric, liver and lung cancer [34], its prometastatic effects in PC are independent of MET activation according to our study. In the present study, MACC1 treatment did not alter the expression levels of MET. Moreover, the selective inhibitor of MET could not reverse the promigratory and proinvasive effects of MACC1, which suggested that MACC1 facilitates PC metastasis in a MET-independent way. Inconsistent with our study, a previous study reported that circ-PDE8A serves as a sponge for miR-338 to upregulate MACC1 and activate the MET or AKT pathways, promoting PC progression [35]. The possible reason for the seemingly inconsistent findings might be that circ-PDE8A might bypass MACC1 to activate MET. Thus, their results are insufficient to prove that MACC1 promotes PC progression in a MET-dependent manner. The exact reason why MACC1 does not promote metastasis via MET in pancreatic cancer is likely complex and deserves further analysis. We postulate that such a phenomenon could be explained by the following reasons. First, the SP1 motif is necessary for MACC1-mediated MET expression in colon cancer cells [31], which suggests that MACC1 regulation of MET requires involvement with SP1; in terms of this, MACC1 may not interact with SP1 and therefore does not regulate pancreatic cancer metastasis via the MET pathway. Second, epigenetic modifications play a vital role in regulating gene expression by altering the structure of the genome, thereby affecting the accessibility of transcription factors and RNA polymerases binding to target genes [36]. For example, alterations in the genomic architecture of MET can interfere with the binding of transcription factors to it [37]. This raises the possibility that epigenetic modifications of MET may lead to the result that MACC1 cannot transcriptionally regulate MET in PC. Third, MACC1 is not the only regulator of MET, and there are many proteins other than MACC1 that can directly regulate MET expression (e.g., HIF1, SP1, AP1, and Ets-1) [38–41], suggesting that MACC1 may not be a predominant regulator of MET in pancreatic cancer. Similar to our result, Sueta A et al. reported that MACC1 cannot activate MET and that MACC1 is not a major regulator of MET in breast cancer [42]. This implies that the same molecule with diverse biological functions depends mainly on distinct biological contexts.

FN1, one of the most abundant glycoproteins in the ECM, is expressed not only in the stroma but also in tumor cells and exerts a significant role in tumor progression, invasion and premetastatic and metastatic disease mainly through integrin-mediated signaling [43–45]. Ectopically overexpressing MACC1 in PC cells resulted in the upregulation of FN1, and downregulating MACC1 in PC cells with high endogenous MACC1 expression also decreased FN1 expression. Furthermore, an in vitro experiment confirmed that FN1 is essential for MACC1-promoted metastasis, and combining the expression levels of MACC1 and FN1 could be used as an improved prognostic indicator for PC. To investigate the underlying molecular mechanisms by which MACC1 regulates FN1 expression, we performed a Co-IP analysis to search for the transcription factors responsible for MACC1-induced FN1 expression. We further identified the EMT regulator SNAI1 as an interactor with MACC1 from seven transcription factors that were previously reported to bind to the promoter region of *FN1*. In parallel, we also observed reduced expression of *CDH1*. SNAI1, a key transcriptional activator of EMT, as well as a critical transcriptional repressor of *CDH1* [46]. Next, using a dual-luciferase reporter assay, we confirmed that MACC1 upregulates *FN1* expression and downregulates *CDH1* expression via promoting SNAI1 transcriptional activity. However, the precise mechanism by which MACC1 upregulates the transcriptional activity of SNAI1 requires further investigation. Previous literature reported that CREB-binding protein (CBP)-mediated acetylation of Snail promotes the transcriptional activity of Snail [32]. Whether MACC1 promotes SNAI1 transcriptional activity by regulating its acetylation needs further study.

**Fig. 7:**
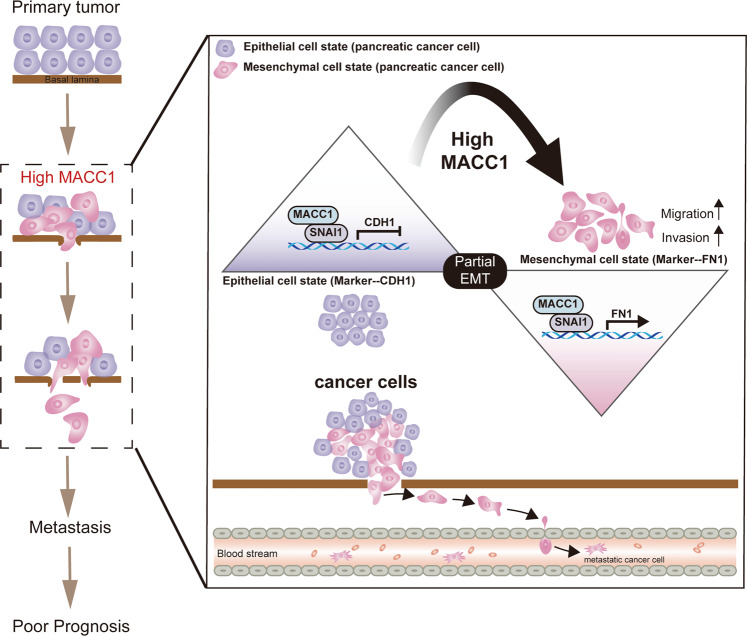
Proposed model for the role of MACC1 on PC metastasis. MACC1 functions as an EMT driver by interacting with SNAI1 to promote the transition of primary cancer cells from the epithelial to the mesenchymal cell state, which drived the EMT program and accelerated the metastasis of pancreatic cancer, and ultimately influenced the patient outcome. On the one hand, the MACC1-SNAI1 complex binds to the CDH1 promoter, inhibits the expression of the epithelial cell marker CDH1, and promotes the loss of the epithelial phenotype of cancer cells. On the other hand, the MACC1-SNAI1 complex binds to the FN1 promoter and promotes the expression of the mesenchymal marker FN1, which further facilitates the transition of cancer cells to the mesenchymal cell state and ultimately accelerated the metastasis of pancreatic cancer.

Overall, our study sheds light on the mechanism by which MACC1 enhances the metastatic ability of PC cells in a MET-independent manner. MACC1 increases SNAI1 transcriptional activity, thus activating *FN1* and repressing *CDH1* (Fig. 7). Furthermore, MACC1 alone or in conjunction with FN1 might serve as a novel biomarker for PC prognosis and targeting the MACC1-SNAI1 complex might represent a promising method for PC metastasis.

## Materials and methods

### Human tissue microarray (TMA) and immunohistochemistry (IHC)

Human PC TMAs were purchased from Outdo Biochip (Shanghai, China); pathologic information and patient survival information were provided. Briefly, paraffin-embedded TMAs were dewaxed in xylene, rehydrated through graded alcohols, and then incubated with anti-MACC1 (Ominimabs, OM283988, 1:200 dilution) and anti-FN1 antibodies (Cell Signaling Technologies, 26836, 1:100 dilution). Two independent clinical pathologists who were blinded to the TMA data assessed the immunostaining of each core according to a standard classification scheme under a light microscope. The final immunohistochemical scoring of MACC1 and FN1 expression was obtained from the histochemical score (H-score). The H-score analysis was calculated as previously described [47].The percentage of positive cells was recorded as follows: 0 (<5%), 1 (5–25%), 2 (26–50%), 3 (51–75%) and 4 (>75%). The staining intensity was recorded from 0 (negative) to 3 (strong).

### Cell culture and chemical reagents

BxPC-3, PANC-1, PATU-8988, SUIT-2, HPDE, SW620 and SW480 cells were provided by the Cell Bank of Chinese Academy of Sciences (Shanghai, China) and grown in RPMI-1640 medium (HyClone) or DMEM medium (HyClone) with 10% fetal calf serum (FCS), 0.1 mg/ml streptomycin and 100 U/ml penicillin. The human embryonic kidney 293 T (HEK293T) cell line obtained from American Type Culture Collection (ATCC) was grown in DMEM (HyClone) with 10% FCS, 0.1 mg/ml streptomycin and 100 U/ml penicillin. The MET kinase inhibitor JNJ-38877605 (Selleck Chemicals, S1114) was dissolved in dimethyl sulfoxide at 50 µM and then diluted 1000-fold with culture medium to obtain a final concentration of 50 nM.

### Plasmids and antibodies

Human full-length *ATF3*, *CREB1*, *EGR1*, *HMGA2*, *HSF1*, *LEF1* and *SNAI1* were subcloned into a pcDNA3.1-HA-C vector. Full-length human *MACC1* was subcloned into the pcDNA3.1-3✖▯FLAG-C vector. The shRNA constructs targeting human *FN1* and *SNAI1* were subcloned into the pLVX-shRNA2-Neo vector respectively. The shRNA constructs targeting human *MACC1* were in the pLVSHG01 vector and were purchased from Cyagen Biosciences (Guangzhou, China). The shRNA target sequences are listed in Supplementary Table 2. The human promoter regions of *FN1* (-2000 bp to +200 bp) and *CDH1* (-200 bp to +100 bp) were cloned into the pGL3 luciferase vector respectively. All constructs were confirmed by DNA sequencing. Transfection was performed using Lipofectamine 2000 (Invitrogen) following the manufacturer’s instructions.

Rabbit monoclonal anti-MACC1 (#86290), anti-phospho-Met (#3077), anti-Snail (#3879), anti-E-Cadherin (#3195) and anti-FN1 (#26836) antibodies were purchased from Cell Signaling Technology (CST). Rabbit monoclonal anti-Met (ab51067) was purchased from Abcam. Rabbit polyclonal anti-PTGS2 (A1253), anti-MMP10 (A3033) and anti-HMGA2 (A2972) antibodies were purchased from Abclone (Wuhan, China). Rabbit polyclonal anti-HA antibody (51064-2-AP, 1:3000), rabbit antibody against the DYKDDDDK tag (20543-1-AP, 1:3000), goat anti-rabbit (SA00001-2) and anti-mouse (SA00001-1) HRP-conjugated secondary antibodies were purchased from Proteintech. Mouse anti-Flag M2 affinity gels (A2220) and anti-HA agarose beads (A2095) were purchased from Sigma. For immunoblotting analysis, primary antibodies were diluted at 1:1,000 except where noted, and secondary antibodies were used at 1:5,000 dilution. For immunofluorescence analysis of PC cells, antibodies against FN1 were used at 1:400 dilution.

### Virus packaging and infection

To generate stable cell lines expressing human MACC1- and FN1-specific shRNAs, HEK293T cells were transfected using Lipofectamine 2000 with the appropriate lentiviral expression vector (encoding MACC1-shRNA, FN1-shRNA or a scrambled control shRNA) and psPAX2 and pMD2.G packaging plasmids as previously described [48]. The harvests of individual supernatants containing recombinant lentivirus were collected 48 hours and passed through a 0.45-μm filter (Millipore). Finally, the collected lentivirus was centrifuged and utilized for infection. For lentiviral shRNA infection, the cells were infected with various lentivirus-containing media and selected with G418 and/or puromycin. MACC1 overexpression lentiviruses were purchased from GeneChem (Shanghai, China). Cells were infected with lentivirus for 24 h and subjected to puromycin selection for at least one week. To obtain stable cell lines expressing cDNA for FN1, a CRISPR-mediated activation system was used for transcriptional activation of FN1, the virus package and the infection method, as previously described [49, 50]. LentiMS2-P65-HSF1_Hygro (plasmid 61426-LV) and lentiSAMv2 (plasmid 75112) were from Addgene. The sgRNA sequences for FN1 are listed in Supplementary Table 2. The sgRNA was designed using the CRISPR design tool (http://crispr-era.stanford.edu). All constructs were confirmed by DNA sequencing.

### Quantitative real-time PCR (qRT–PCR)

Total RNA extraction, cDNA synthesis and quantitative RT–PCR were performed as described before [51]. The primers are listed in Supplementary Table 2.

### Immunoprecipitation and immunoblotting

For exogenous coimmunoprecipitation, 293 T cells seeded in 10 cm Petri dishes were lysed in 400 µl EBC buffer (50 mM Tris, pH 7.5, 120 mM NaCl, 0.5% NP-40) supplemented with protease inhibitors (Roche, 04693159001) and phosphatase inhibitors (Beyotime, P1081). The 400 µl lysate supernatant was divided into 160 µl Flag-IP group, 160 µl HA-IP group and 80 ul Input group. For the IP groups, 8 µl anti-Flag beads and 8 µl anti-HA beads were added to the Flag-IP group and HA-IP group, respectively, and incubated for 3-4 h at 4 °C. After incubation, the IP products were washed three times in NETN buffer(100 mM NaCl, 20 mM Tris pH 8.0, 1 mM EDTA, 0.5% NP-40) and resolved in 35 ul 3x loading buffer for immunoblotting. For the input groups, add 40 µl 3x loading buffer was added into 80 µl lysates for immunoblotting. Immunoblotting assays were performed as previously described [52].

### Human metastasis qPCR array

The human metastasis qPCR array was performed by BioWavelet (Chongqing, China) AutoArray (AA-H0010) with a standard procedure. The gene list obtained by the qPCR array was analyzed using R, version 3.6.2.

### Human tissue samples

Human PC tissues and corresponding normal tissues were provided by Southwest Hospital Affiliated to Army Medical University (Chongqing, China). All patients signed informed consent forms prior to the study. All specimens were immediately frozen in liquid nitrogen and stored at −80 °C until use.

### Mouse models of PC cell metastasis

Ethical approval for all animal experiments was granted by the Guangxi Medical University (Nanning, China) animal ethics committee. Male nude mice (3–4 weeks old) were anesthetized with 50 mg/kg sodium pentobarbital through intraperitoneal injection. The abdomen below the left costal margin was shaved and disinfected with iodine solution, and then a 0.5-1 cm incision was made to expose the abdominal cavity. For intrasplenic injections of pancreatic cancer cells (PANC-1 and BxPC-3 cell lines), cell suspensions (1 × 106 pancreatic cancer cells in 200 µl PBS) were prepared and slowly injected into the exteriorized spleen via a 32-gauge needle. After injection, the spleen was removed from connective and vascular tissues via local accurate cauterization. After splenectomy, the connective and vascular tissues were placed back into the abdominal cavity. Afterward, the peritoneum and skin were sutured respectively. After one month, the mice were euthanized to assess tumor metastasis in the liver by photographing, weighing and hematoxylin and eosin (HE) staining.

### Statistical analysis

Statistical analyses were conducted using GraphPad Prism 7.0 software. Statistical significance was assessed by two-tailed Student’s t test, Mann–Whitney test, one-way ANOVA or two-way ANOVA, as appropriate. The results are displayed as the mean ± SD, unless stated otherwise. Receiver operating characteristic (ROC) curves were applied to find the optimal cut point. Pearson correlation analysis was applied to assess the relationship between two variables. Survival analysis was performed using the log-rank test. Statistical significance was designated as asterisks: *p < 0.05, **p < 0.01, ***p < 0.001 and ****p < 0.0001; ns for not significant.

## Supplementary information

supplementary table 1

supplementary table 2

SUPPLEMENTAL MATERIAL

original western bolts

Reproducibility Checklist

## Data Availability

Data will be made available on reasonable request.
