# Supplementary material for: MACC1 promotes pancreatic cancer metastasis by interacting with the EMT regulator SNAI1
Source: Cell Death Dis. 2022 Nov 4;13(11):923. doi: 10.1038/s41419-022-05285-8 (PMC9636131; doi:10.1038/s41419-022-05285-8)
Supplement: Supplementary file 3 — SUPPLEMENTAL MATERIAL [file 41419_2022_5285_MOESM3_ESM.pdf]

Supplementary Methods

Clonogenic assay

Pancreatic cells were digested into a single cell state at a density of 1 × 104, and then 100 μl cell suspensions were seeded in a six-well plate loaded with 1.9 ml complete medium to culture for 2-3 weeks. Following visible cell clone formation, cells were prepared as in the Migration and Invasion Assay.

Cell proliferation assay

Cell proliferation was examined using a Cell Counting Kit-8 (Beyotime, C0043). Briefly, cell suspensions (2 × 10^3/well) were seeded in 96-well culture plates and incubated for 3 days. CCK8 solution (10 μL) was added to each well, and the cells were cultured for another 2 h. The optical density was measured at 450 nm using a microplate reader.

Invasion and migration assays

For the invasion assays, cells were plated on Matrigel (Corning, 356234)-coated Transwell chambers (Millipore, MCEP24H48). For the migration assays, the Matrigel coating step was not performed. In both assays, cells were precultured in a serum-free environment for 12 h, and equal numbers of cells were plated on each Transwell chamber in serum-free medium. Cells were incubated for 24 h (migration) or 48 h (invasion). After gentle removal of nonmigrated cells on the upper side of the filter by a cotton swab, the chambers were fixed with 4% paraformaldehyde for 20 min and stained

with 0.1% crystal violet for 15-20 min. Afterward, the chambers were washed several times in deionized water and air-dried for photographing by an inverted microscope (Olympus) and counting by ImageJ.

JNJ-38877605 inhibition assay

Cells were resuspended in serum-free medium (supplemented with JNJ-38877605 or DMSO) and cultured in the corresponding chamber, followed by 24 h (migration) or 48 h (invasion) incubation. Cells were prepared as in the Migration and Invasion Assay.

Wound-healing assay

In 6-well plates, cells were seeded and serum-starved overnight when they reached confluence. The following day, the monolayer of confluent cells was scratched with a 200 μl pipette tip. Next, the cells were grown under serum-free conditions and photographed at designated time points under an inverted microscope (Olympus). The wound area was measured in ImageJ.

Immunofluorescence (IF)

Pancreatic cancer cells were grown in glass-bottom cell culture dishes (NEST, 801101) for 48 h and fixed with 4% formaldehyde for 15 min at RT. The culture dishes were rinsed three times in PBS for 5 min each and immunostained. Specimens were blocked in blocking buffer (10% goat serum in PBS supplemented with 0.2% Triton X-100) for 60 min at RT and then incubated overnight at 4°C in FN1 primary antibody diluted 400-

fold in primary antibody dilution buffer (Zhongshan Golden Bridge Biotechnology, ZLI9030). After incubation, the culture dishes were rinsed and incubated in AF555-conjugated secondary antibody (Invitrogen, A-31572) diluted 600-fold in secondary antibody dilution buffer(Beyotime, P0108) for 40 min at 37°C. After immunostaining, the culture dishes were rinsed and incubated in DAPI (Beyotime, C1005) for 3-5 min at RT in the dark. Afterward, the culture dishes were rinsed and sealed with antifading mounting medium (Solarbio, S2100). Images were acquired using fluorescence microscopy (Olympus).

Luciferase reporter assay

The promoter activity was detected according to a standard protocol (Promega, E1910). The pRL-TK plasmid was used as a transfection control. All values were normalized for transfection efficiency against Renilla luciferase expression.

FACS analysis for apoptosis

After ectopically overexpressing MACC1 in the PANC-1 and SUIT-2 cell lines and downregulating MACC1 in the BxPC-3 cell line, an APC Annexin V Apoptosis Detection Kit (Biolegend, 640932) was used for apoptosis and necrosis analysis according to the manufacturer’s instructions.

Subcutaneous tumor xenograft model of PC cells

For the subcutaneous tumor xenograft model, 2×10^6 PANC-1 and MACC1-upregulated PANC-1 cells (or BxPC-3 and MACC1-downregulated BxPC-3 cells) were resuspended

in 100 μl PBS and subcutaneously inoculated into the left armpit and right armpit, respectively. After one month, the mice were euthanized to assess the tumor size by photographing and weighing.

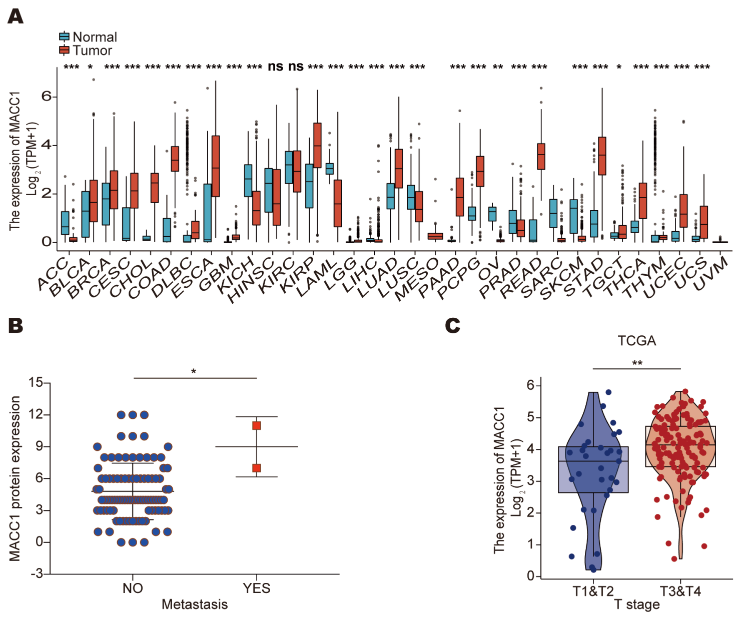

Fig. S1. Clinical characteristics correlated with MACC1 levels. (A) Expression levels of *MACC1* in the pancancer TCGA dataset. *p < 0.05, **p < 0.01, ***p < 0.001. ns for not significant. **(B)** MACC1 protein expression levels of PC samples grouped by metastasis (data from TMA). Two-tailed t test. *p < 0.05. **(C)** MACC1 mRNA expression levels of PC samples grouped by T stage (data retrieved from the TCGA database).Two-tailed t test. **p < 0.01.

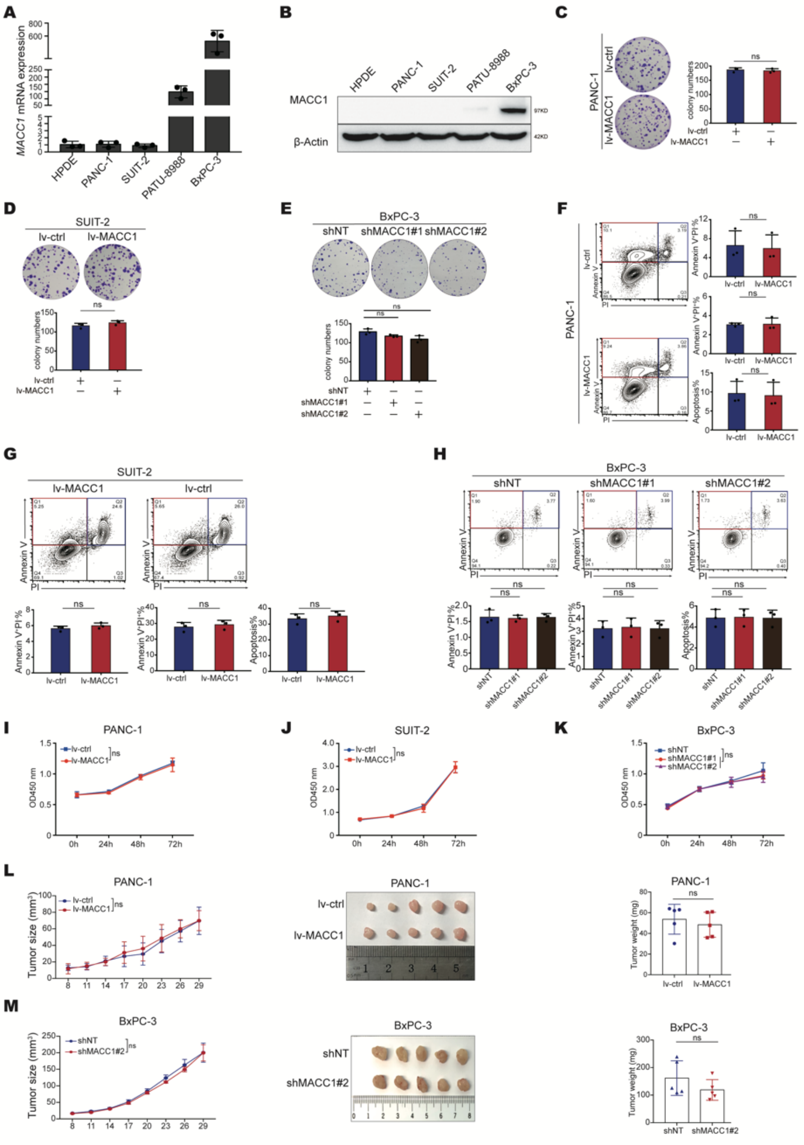

Fig. S2. MACC1 did not affect the proliferation and apoptosis of PC cells. (A-B) qRT–PCR and western blot analysis of MACC1 expression in normal pancreatic ductal epithelial cells (HPDE) and PC cell lines (PANC-1, SUIT-2, PATU-8988, BxPC-3). **(C-E)** The effect of MACC1 overexpression in PANC-1 and SUIT-2 cells or knockdown in BxPC-3 cells **(E)** on clonogenicity. ns for not significant. **(F-H)** The effect of MACC1 overexpression in PANC-1 and SUIT-2 cells or knockdown in BxPC-3 cells **(H)** on apoptosis. ns for not significant. **(I-K)** The effect of MACC1 overexpression in PANC-1

and SUIT-2 cells or knockdown in BxPC-3 cells **(K)** on proliferation. ns for not significant. **(L)**Growth curve (left), tumor size (middle) and tumor weight (right) of mice subcutaneously inoculated with PANC-1 cells expressing MACC1 (lv-MACC1) or empty vector(lv-ctrl) (mean ± SD, five mice per group). Growth curve: two-way ANOVA. Tumor weight: two-tailed t test. **(M)** Growth curve (left), tumor size (middle) and tumor weight (right) of mice subcutaneously injected with BxPC-3 cells expressing shMACC1#2 or the corresponding control (shNT) (mean ± SD, five mice per group). Growth curve: two-way ANOVA. Tumor weight: two-tailed t test. **C-D**, **F**, I-J, Data represent the mean ± SD. of three biologically independent experiments (two-tailed t test). ns for not significant. **E**, **H**, K, Data represent the mean ± SD. of three biologically independent experiments (one-way ANOVA).

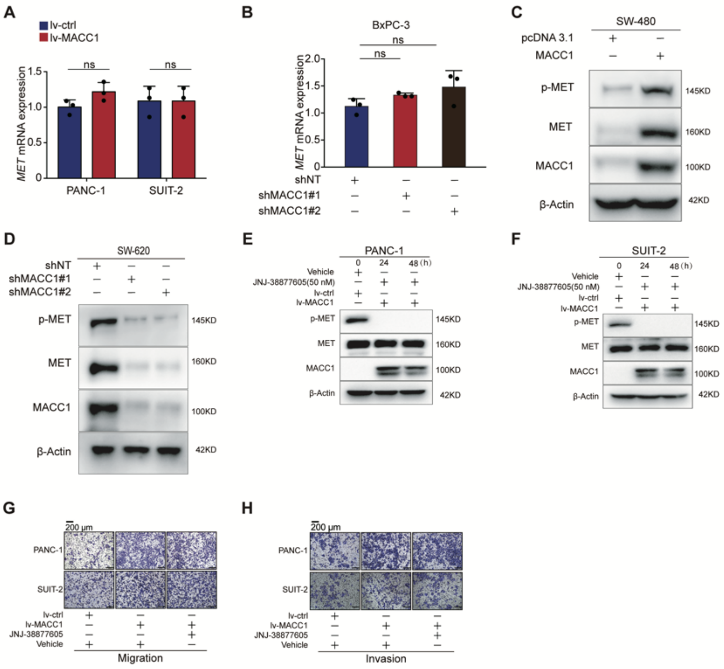

Fig. S3. The role of MACC1 in PC is different from that in colon cancer. (A-B) The effect of MACC1 overexpression in PANC-1 and SUIT-2 cells or knockdown in BxPC-3 cells **(B)** on MET mRNA levels. Data are shown as the mean ± SD. **A**: two-tailed t test. **B**: One-way ANOVA. ns for not significant. **(C-D)** The effect of MACC1 overexpression in SW-480 cells or knockdown in SW-620 cells on p-MET and MET protein levels. **(E-F)** MACC1 and SUIT-2 cells (with stable overexpression of MACC1 or empty vector) were treated with 50 nM JNJ-38877605 (a selective inhibitor of MET) or vehicle (0.1% DMSO) and IB was performed to determine the effect on MET activation. **(G-H)** Migration and invasion of PANC-1 and SUIT-2 cells treated with 50 nM JNJ-38877605 and an equal volume of DMSO treatment was used as a vehicle control. Representative images of the migrated and invaded cells are shown (mean ± SD, n = 5). Two-way ANOVA. Scale bars, 200 μm. Data are representative of at least three independent experiments.

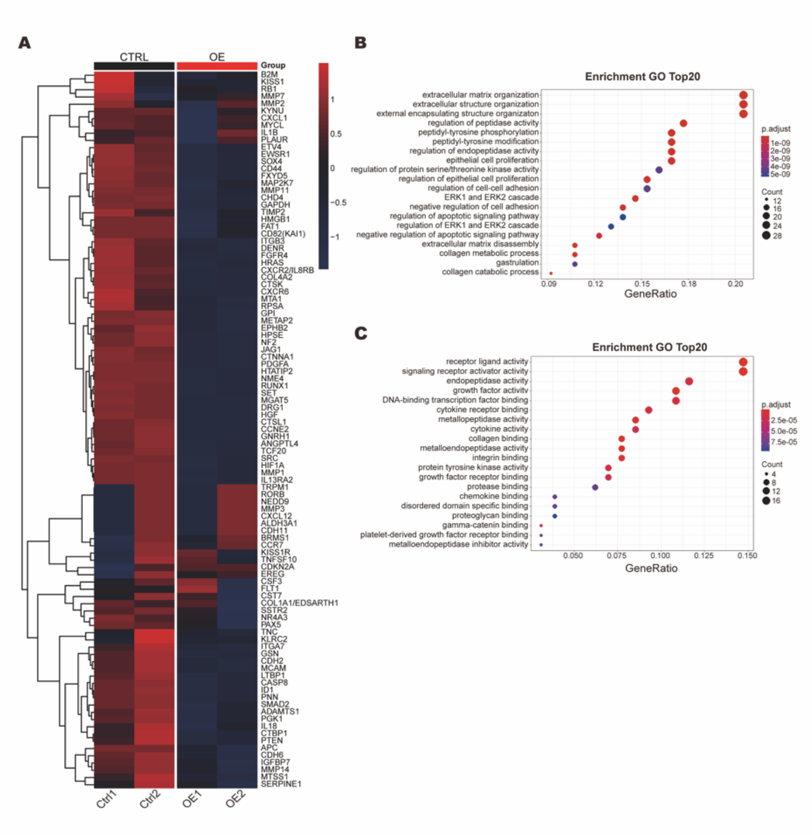

Fig. S4. Bioinformatic analysis of the MACC1-regulated genes. (A) Heatmap of the downregulated gene expression clusters in control (Ctrl) and MACC1-overexpressing (OE) SUIT-2 cells. **(B)** GO analysis of biological processes for the MACC1-regulated genes. **(C)** GO analysis of molecular functions for the MACC1-regulated genes.

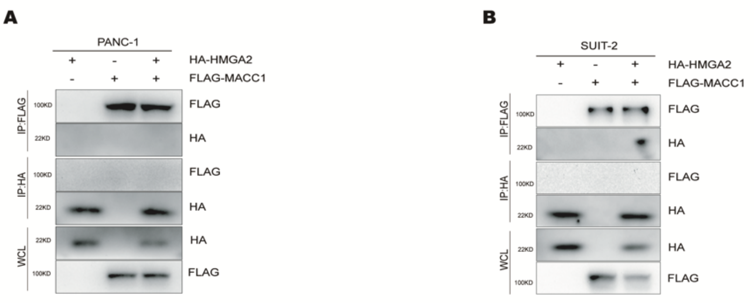

Fig. S5. MACC1 does not interact with HMGA2 in PC cells. (A) Coimmunoprecipitation of exogenous MACC1 and HMGA2 in PANC-1 cells. **(B)**

Coimmunoprecipitation of exogenous MACC1 and HMGA2 in SUIT-2 cells. Data are representative of at least three independent experiments.

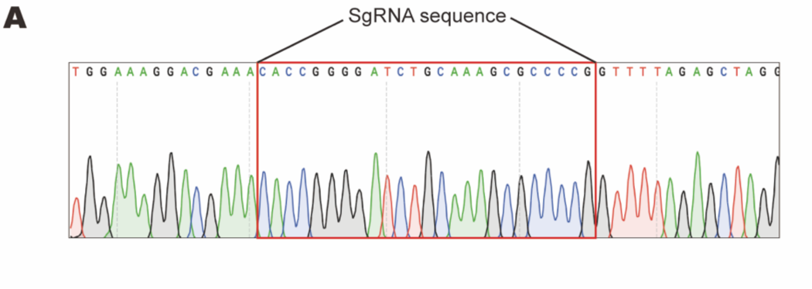

Fig. S6. FN1 sgRNA Sequence. (A) Sequencing results of the FN1 promoter locus targeted by sgRNA.

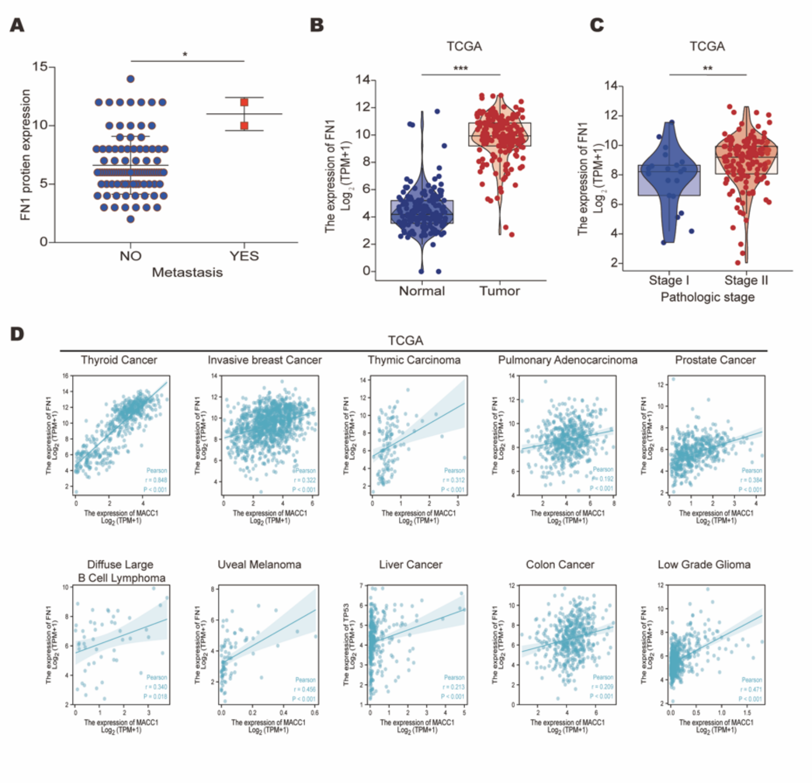

Fig. S7. Clinical characteristics correlated with FN1 levels. (A) FN1 protein expression levels of PC samples grouped by metastasis (data from TMA). Two-tailed t

test. *$p < 0.05$. **(B)** The *FN1* expression in TCGA PC and normal tissues. Two-tailed t test. ***$p < 0.001$. **(C)** *FN1* mRNA expression levels of PC samples grouped by pathologic stage (data retrieved from TCGA database). Two-tailed t test. **$p < 0.01$. **(D)** The correlation of the mRNA expression levels between *MACC1* and *FN1* in 10 cancer types from the TCGA datasets. R, Pearson correlation coefficient.
