# Supplementary figures and images for: MACC1 promotes pancreatic cancer metastasis by interacting with the EMT regulator SNAI1
Source: Cell Death Dis. 2022 Nov 4;13(11):923. doi: 10.1038/s41419-022-05285-8 (PMC9636131; doi:10.1038/s41419-022-05285-8)

Original full length western blots

Fig. 2

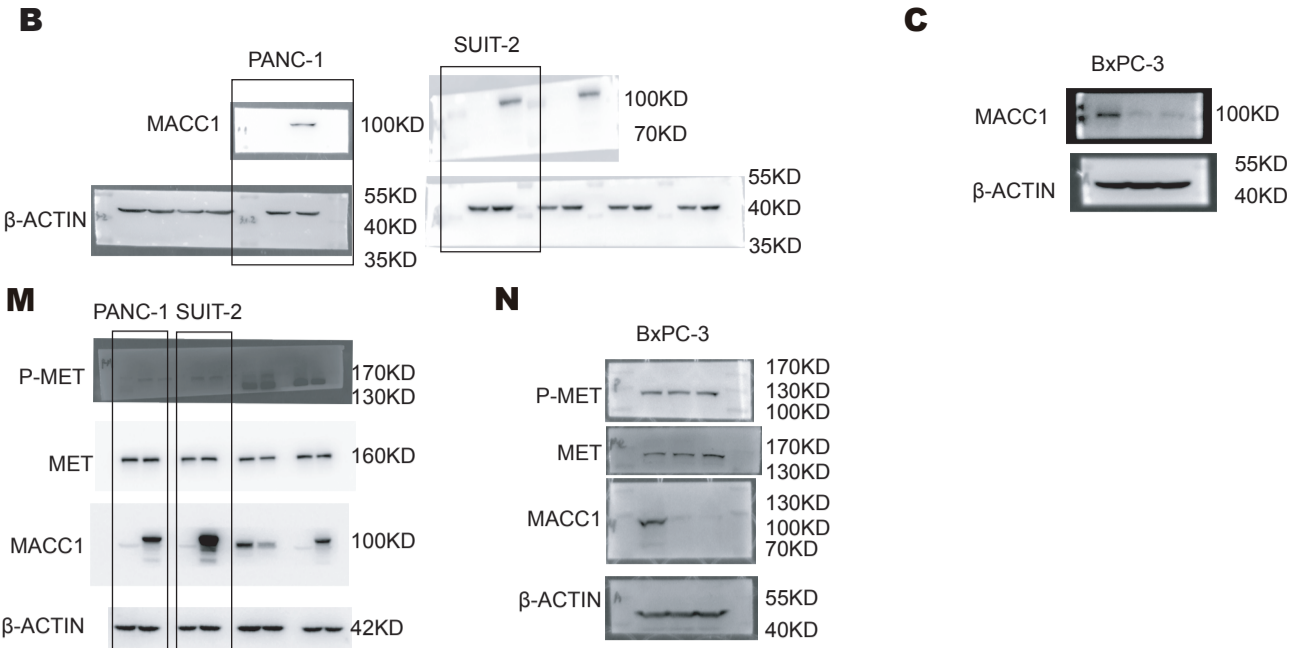

Fig. 3

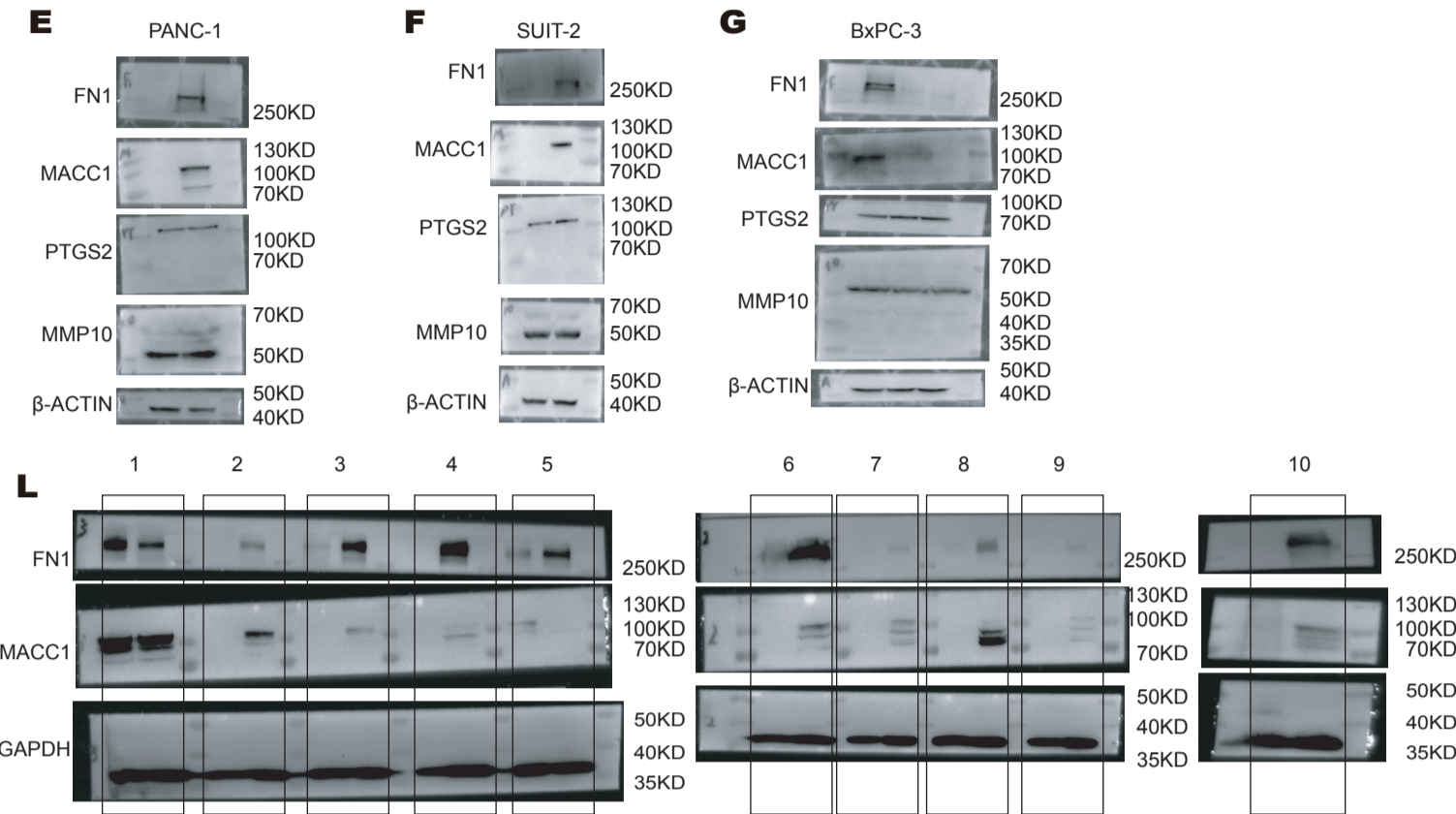

Fig. 4

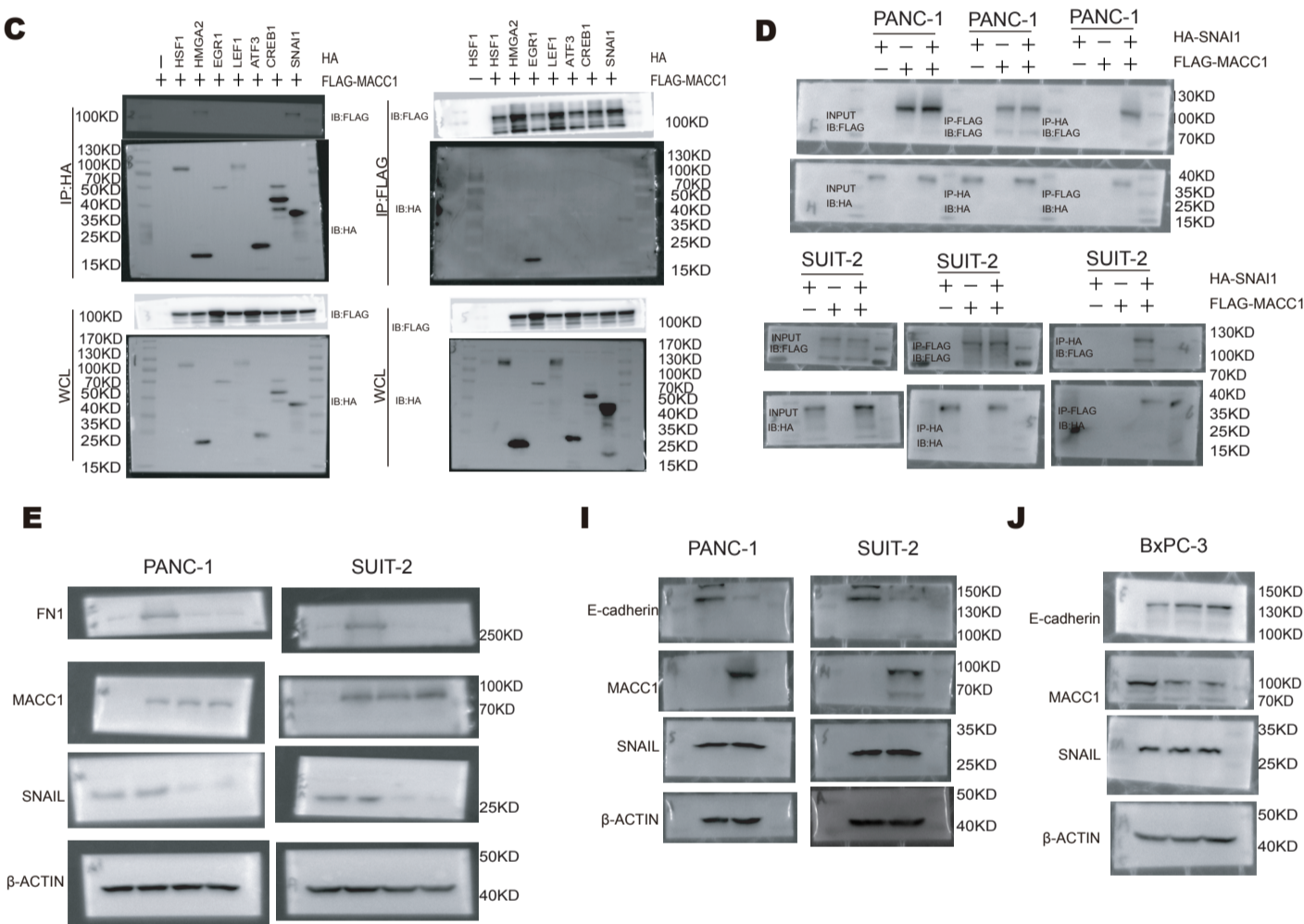

Fig. 5

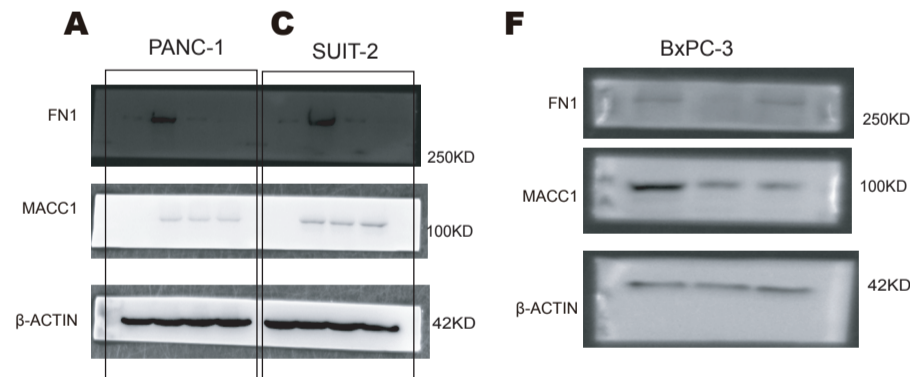

补充数据

Fig. S2

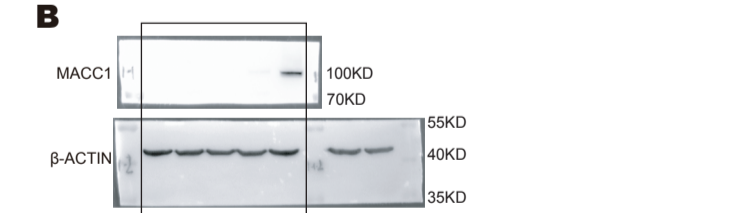

Fig. S3

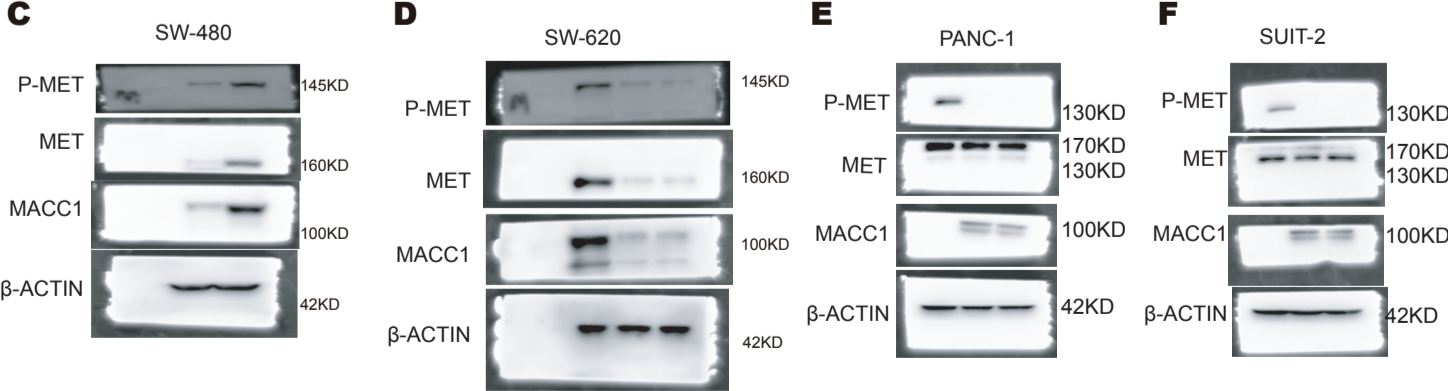

Fig. S4

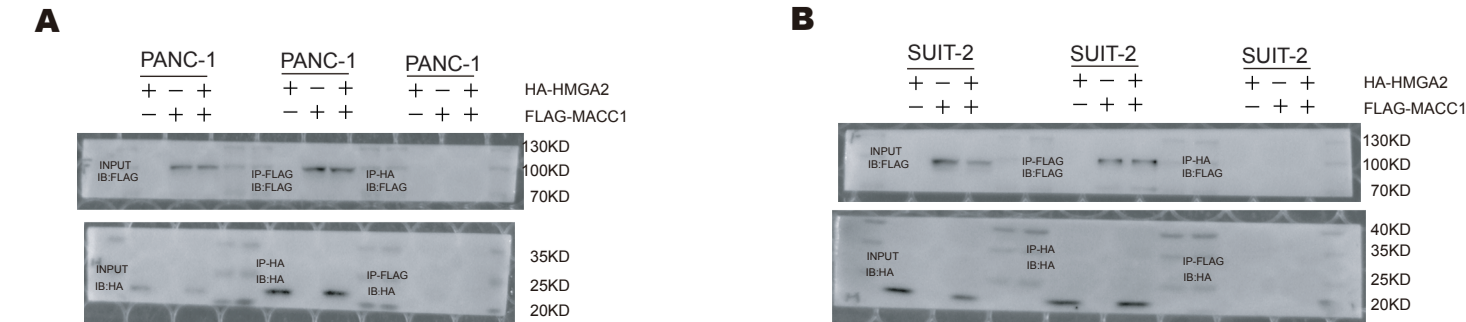

Supplement: Supplementary file 4 — original western bolts [file 41419_2022_5285_MOESM4_ESM.pdf]
